# Piperacillin–tazobactam tolerability in patients with a labeled penicillin allergy (PIPPEN)

**DOI:** 10.1017/ash.2026.10322

**Published:** 2026-03-27

**Authors:** Avneet Purewal, Kieran Shah, Sarah N. Stabler, Kevin Afra, Ke Su

**Affiliations:** 1 Fraser Health Authority, Canada; 2 The University of British Columbia, Canada

## Abstract

**Background:**

Piperacillin–tazobactam is a beta-lactam antibiotic with a distinct R1 side chain compared to other penicillins, therefore the risk of cross-reactivity should be theoretically low. Despite this difference, antimicrobial guidelines recommend against using piperacillin–tazobactam in patients with penicillin allergies. There is limited data available regarding piperacillin–tazobactam use in patients with a penicillin allergy, or rates of cross-reactivity. Therefore, the objective of this study was to assess the incidence of cross-reactivity to piperacillin–tazobactam in patients with a labeled penicillin allergy.

**Methods:**

This was a retrospective study of patients admitted to Surrey Memorial Hospital (SMH) between November 1^st^, 2021 to January 31^st^, 2024 assessing tolerance to piperacillin–tazobactam in patients with prior labeled penicillin allergies. Baseline characteristics and outcomes were collected from electronic medical records (EMR).

**Results:**

Of the 191 patients included, 98% were found to tolerate one or more doses of piperacillin–tazobactam. This included 95 patients with “low risk delayed reactions,” 90 patients with “high risk anaphylactic reactions,” and 2 patients with “well-documented delayed reactions,” to penicillins. Only four patients out of 191 had documented intolerance to piperacillin-tazobactam post-exposure.

**Conclusion:**

This study suggests that piperacillin–tazobactam has a low risk of intolerability (2%) in patients with labeled penicillin allergies, and that it is reasonable to consider piperacillin–tazobactam as an alternative to carbapenems and other broad-spectrum antibiotics for most patients with a previous penicillin allergy.

## Introduction and background

Piperacillin–tazobactam is a combination antibiotic containing a semi-synthetic extended-spectrum ureidopenicillin (piperacillin) and a B-lactamase inhibitor (tazobactam).^1^ Classified as a penicillin antibiotic, it is one of the few antimicrobials that has coverage against *Pseudomonas aeruginosa*, along with gram-positive and gram-negative bacteria.^2^ Due to its anti-pseudomonal coverage, it is an excellent choice for hospital-acquired infections and cephalosporin-resistant infections. However, its use can be limited due to the high prevalence of penicillin allergies.^3^

Prevalence of beta-lactam antibiotic allergies, including penicillins, cephalosporins, and carbapenems, has been reported to be around 5%–10% in previous literature and is the most commonly reported medication allergy.^3–6^ This class of antibiotics contain structural similarities which are responsible for the allergic cross-reactivity which occurs amongst them.^6^ Originally it was thought that immunoglobulin E (IgE) mediated hypersensitivity to beta-lactams occurred from the similar B-lactam ring structure; however current literature demonstrates that the R1 side chains, and less commonly the R2, are responsible for the cross-reactivity.^4,7,8^ For this reason, it is recommended that patients allergic to one penicillin antibiotic should avoid all other penicillins and cephalosporins with similar R1 side chains.^9,10^ However, despite its distinct side chain, current guidelines recommend avoiding piperacillin–tazobactam in patients with allergies to other penicillin antibiotics.^9,10^

Few studies to date have explored the relationship between piperacillin–tazobactam and penicillin allergies, most of which largely center around penicillin skin testing. In a study by Wong et al. (2021), the cross-reactivity of penicillin and piperacillin–tazobactam was examined in 11 patients with verified piperacillin–tazobactam allergies, and it was found that only 1 patient had a positive skin test for a penicillin allergy.^2^ In a similar study, Gallardo et al. (2020) described 10 patients with verified piperacillin–tazobactam allergies and found 3 patients had a positive skin test to either amoxicillin or penicillin, and only 1 patient had a positive skin test to both amoxicillin and penicillin.^11^ Comparably, a study by Casimir-Brown et al. (2021) found that patients sensitive to piperacillin–tazobactam were able to tolerate other penicillins on skin testing.^1^ Of 24 patients with a verified piperacillin allergy, 16 patients were sensitive to piperacillin only, and showed no cross-reactivity to the other penicillins. Of the eight patients that did experience cross-reactivity, each patient displayed a unique pattern to their skin tests, meaning not all reacted to the same penicillin antibiotic. The study also identified that tazobactam may also be a source of potential allergy in patients who have a hypersensitivity to piperacillin–tazobactam, however this needs to be studied further. Although limited by their small sample size, the three studies highlighted low cross-reactivity between penicillin and piperacillin–tazobactam.

Currently, there is insufficient clinical evidence to determine cross-reactivity between piperacillin–tazobactam and other penicillin antibiotics. Some guidelines may recommend avoiding piperacillin–tazobactam in patients with penicillin allergies or may not address the issue due to insufficient evidence regarding cross-reactivity with penicillins. The Drug Allergy: A 2022 Practice Parameter Update guideline by Khan et al. (2022) acknowledges that recent reports have shown that patients may have selective allergies to piperacillin–tazobactam, and these patients can tolerate other penicillins despite a piperacillin–tazobactam allergy, however no formal guidance has been provided.^12^ Therefore, due to a lack of specific guidance for piperacillin–tazobactam use in penicillin allergic patients, this has resulted in carbapenems typically being the antibiotic of choice in penicillin allergic patients requiring broad-spectrum antibiotics. However, with carbapenem resistance increasing in Canada and worldwide, other treatment options need to be considered.^10^ Piperacillin–tazobactam remains an effective option against *P. aeruginosa* and therefore it could potentially reduce carbapenem usage.^14,15^ No study to date has directly assessed the safety of piperacillin–tazobactam in patients with a labeled penicillin allergy, as the current literature is limited to skin testing studies.^1,2,11^ Therefore, this study was designed to assess the tolerability of piperacillin–tazobactam in patients with labeled penicillin allergies.

## Methods

### Study design

This study was a retrospective chart review conducted of the electronic medical records (EMR) at Surrey Memorial Hospital (SMH) from November 1^st^, 2021 to January 31^st^, 2024. Patients were included if they met the following criteria: greater than or equal to 18 years old, documented penicillin allergy prior to admission, and received at least one dose of piperacillin–tazobactam during admission. Patients were excluded if they had a previous successful penicillin skin test or a successful penicillin challenge prior to piperacillin–tazobactam dose administration. Challenges were considered successful if the patient tolerated the penicillin without an allergic reaction. Patients were also excluded if they had a penicillin intolerance label secondary to a non-allergy-related adverse drug reaction (e.g., nausea or diarrhea). Readmissions during the study period were excluded to prevent multiple events for one participant. Lastly, patients were excluded from the study if they were discharged within 24 hours of their first dose of piperacillin–tazobactam due to inability to accurately assess tolerability.

After approval for the study through the Fraser Health Research Ethics Board (FHREB) and The University of British Columbia Researcher Information Services (RISe), patients with a penicillin allergy who received piperacillin–tazobactam during hospital admission were identified using the LUMED antimicrobial stewardship program software (LUMED, Quebec, Canada).

### Data collection

Data was collected from the EMR for the study population. Baseline characteristics, including age, sex, and penicillin antibiotic allergy (including medication, reaction date, type of hypersensitivity reaction, and penicillin allergy risk stratification according to regional antimicrobial stewardship guidelines) were collected (Table 1). Additionally, other non-penicillin antibiotic allergies, use of medications for treatment or prophylaxis of allergies, and critical care ward (Intensive Care Unit (ICU) or High Acuity Unit (HAU) admissions within 24 hours of piperacillin–tazobactam administration were collected. For patients who were intolerant to piperacillin–tazobactam, additional data including time to intolerance, description of the reaction, high allergy risk medications (medications associated with a high risk of allergies, such as antibiotics, anticonvulsants, nonsteroidal anti-inflammatory medications, or chemotherapy medications^16^) within 48 hours before or after piperacillin–tazobactam administration, and any treatment medications given were recorded.

**Table 1.** Fraser Health Authority (FHA) Antimicrobial Stewardship (AMS) risk stratification for penicillin allergies chart^10^

Risk stratification	Description
Low risk delayed reaction (over 72 h)	eg Morbilliform rashes (in absence of pruritus) are not typical of type I reactions Poorly described symptoms (no hospitalization, no systemic involvement, occurred >10 yr ago)
High risk anaphylactic reactions (within 72 h)	eg Angioedema, urticarial/pruritus, laryngeal edema, wheezing, hypotension. Poorly described symptoms but patient has poorly controlled and/or unstable cardiac or respiratory comorbidity.
Well-documented delayed allergic reactions	Impacts all penicillins: Stevens-Johnson syndrome, toxic epidermal necrolysis, drug reaction with eosinophilia or and systemic symptoms (DRESS), immune hepatitis, interstitial nephritis, small vessel vasculitis Drug-specific*: hemolytic anemia, thrombocytopenia, agranulocytosis, neutropenia

*Drug-induced hemolytic anemia, thrombocytopenia, agranulocytosis, and neutropenia are drug-specific. Cross-reactivity between cephalosporins and penicillins does not appear to occur. Avoid the offending drug.

Tolerability of piperacillin–tazobactam was assessed based on physician notes and nursing documentation from the first dose of piperacillin–tazobactam up to a total of 5 days during hospitalization or until discharge, whichever occurred first. This duration was based on the onset of a delayed hypersensitivity reaction in a previously sensitized individual, which can present up to 4 days post-antibiotic administration.^10^

### Outcomes

The primary outcome was the proportion of patients who tolerated piperacillin–tazobactam with a labeled penicillin allergy. Secondary outcomes included a subgroup analysis of the proportion of patients who tolerated piperacillin–tazobactam in different risk stratification groups outlined in the FHA Antimicrobial Stewardship Program’s Risk Stratification for Penicillin Allergies guide (Table 1),^10^ as well as the patients who tolerated piperacillin–tazobactam with a previous anaphylactic or anaphylactic-like (SOB, angioedema, swelling) allergy to penicillins.

### Statistical analysis

Descriptive statistics were used to define the data.

## Results

A total of 191 patients met the inclusion criteria between November 1^st^, 2021 to January 31^st^ 2024. Baseline characteristics of the patient population are summarized in Table 2. The patient cohort had a median age of 67 years old, and 51% were male. Most patients had an unknown amount of time since the index penicillin allergic reaction (73%) and the most common hypersensitivity classification was “unclassified” (72%) which represented undocumented reactions. 98 (51%) patients were classified in the “low risk delayed reactions,” group, 91 (48%) patients were classified in the “high risk anaphylactic reactions or poorly described symptoms and unstable cardiac or respiratory comorbidity,” group, and 2 (1%) patients were classified in the “well-documented delayed reactions,” group. Of note, patients with previous unclassified or unknown reactions admitted to the ICU at the time of the receiving piperacillin–tazobactam were placed in the “high risk anaphylactic group” due to unstable hemodynamics according to the FHA AMS Risk Stratification for Penicillin Allergies guide, which accounts for the higher proportion of patients in this group.

**Table 2.** Baseline characteristics

Characteristic	Number of patients, n=191 (%)
**Male**	97 (51%)
**Female**	94 (49%)
**Age, years (IQR)**	67 (53–78)
**Penicillin allergy^a^**	
** **Penicillin	111 (58%)
“Penicillins”	49 (26%)
Amoxicillin/ampicillin	30 (16%)
Cloxacillin	8 (4%)
**Date of penicillin allergy**	
Unknown	140 (73%)
<1 yr	1 (1%)
1–5 yr	7 (4%)
5–10 yr	3 (2%)
>10 yr	40 (20%)
**Penicillin hypersensitivity classification**	
Type 1	49 (25%)
Type 2	2 (1%)
Type 3	1 (1%)
Type 4	1 (1%)
Unclassified	138 (72%)
**Penicillin allergy risk stratification based on the Fraser Health Authority Antimicrobial Stewardship Handbook**	
Low risk delayed reactions or poorly described symptoms	98 (51%)
High risk of anaphylactic reactions or poorly described symptoms and unstable cardiac or respiratory comorbidity	91 (48%)
Well-documented delayed allergic reactions	2 (1%)
**Number of other antibiotic allergies**	
0	148 (77%)
1	32 (17%)
≥2	11 (6%)
**Penicillin allergy description^b^**	
Unclear/unknown	86
“Rash”	62
Hives/itch	26
Shortness of breath (throat closing, difficulty breathing)	13
Anaphylaxis	7
Angioedema	7
“Swelling”	2
Eosinophilic rash	1
Hypotension	1
Purpuric rash	1
Steven Johnson syndrome	1
**Location of care during hospital admission**	
Critical care ward	54 (28%)
Other wards	137 (72%)

a If patients had more than one penicillin allergy documented, all penicillin allergies were separately counted for the “Penicillin Allergy” section.

b The “Penicillin Allergy Description” section includes a list of all documented reactions to penicillins listed in the patient’s electronic medical record profile. If multiple descriptions were listed, all descriptions were counted and the most severe reaction was classified.

Penicillin allergy descriptions were used to determine risk stratification categories (Table 1), and if a patient had multiple descriptions listed, all descriptions were recorded and the most severe reaction was classified (eg. If anaphylaxis and rash were listed, anaphylaxis was used to determine risk stratification).

29 of the 191 patients in this study had a documented anaphylactic allergy or an anaphylactic-like reaction to penicillin, which included the reactions of “shortness of breath,” “angioedema,” or “swelling” of an unknown area.

For the primary outcome, 187 (98%) of patients in this study tolerated one or more doses of piperacillin–tazobactam (Table 3). All patients in the “anaphylactic allergy or anaphylactic like reaction” subgroup tolerated one or more doses of piperacillin–tazobactam.

**Table 3.** Outcomes; primary outcome: proportion of patients who tolerated piperacillin–tazobactam with a labeled penicillin allergy. Secondary outcome: tolerance based on FHA AMS penicillin allergy risk stratification, proportion of patients with a labeled anaphylactic allergy or anaphylactic like reactions who tolerated piperacillin–tazobactam

Primary outcome		
Tolerance to piperacillin–tazobactam	Number of patients (%)	
Yes	187 (98%)	
No	4 (2%)	

Subgroup analysis: piperacillin-tazobactam tolerance based on FHA AMS penicillin allergy risk stratification		
Penicillin allergy risk stratification based on the Fraser Health Authority Antimicrobial Stewardship handbook	Number of patients who tolerated piperacillin-tazobactam (%)	Number of patients who did not tolerate piperacillin-tazobactam (%)
Low risk delayed reactions or poorly described symptoms (n=98)	95 (97%)	3 (3%)
High risk Of anaphylactic reactions or poorly described symptoms and unstable cardiac or respiratory comorbidity (n=91)	90 (99%)	1 (1%)
Well-documented delayed allergic reactions (n=2)	2 (100%)	0 (0%)

Secondary outcome: proportion of patients with a labeled anaphylactic allergy or anaphylactic like reactions who tolerated piperacillin-tazobactam		
Labeled allergic reaction to penicillin	Number of patients (%)	
Shortness of breath (throat closing, difficulty breathing) (n=13)	13 (100%)	
Anaphylaxis (n=7)	7 (100%)	
Angioedema (n=7)	7 (100%)	
“Swelling” (n=2)	2 (100%)	

As seen in Table 4, 4 patients of 191 did not tolerate piperacillin–tazobactam. Patient A experienced a rash to the face and neck after 15 doses of piperacillin–tazobactam, requiring a dose of epinephrine. Confounding factors at the time of the intolerance event for Patient A included concurrent cefazolin and metronidazole use within 24 hours of piperacillin–tazobactam. Patient A was noted to have previously tolerated piperacillin–tazobactam without an allergic reaction. Patient B experienced right-sided facial swelling and redness after receiving 4 doses of piperacillin–tazobactam, which resolved without treatment. Confounding factors for Patient B included concurrent facial trauma. Patient C had no documentation regarding the intolerance event in hospital, and no treatment medications were provided after receiving piperacillin–tazobactam. The allergic reaction to piperacillin–tazobactam was self-reported on a subsequent admission, and confounding factors at the time of the intolerance event included concurrent cefazolin and metronidazole use within 6 h of piperacillin–tazobactam. Lastly, Patient D reported nonspecific “itchiness” after 1 dose of piperacillin–tazobactam and required one dose of diphenhydramine for treatment. Confounding factors included concurrent cefazolin use within 24 hours of piperacillin–tazobactam. The Naranjo Adverse Drug Reaction Probability Scale (Naranjo) was completed to assess the causality of the intolerance events, and it was calculated that three of the intolerance events were classified as possible adverse drug reactions (ADR), and one was classified as a probable adverse drug reaction.

**Table 4.** Description of piperacillin-tazobactam allergic reaction

Characteristics of the piperacillin-tazobactam allergic reaction	Patient A	Patient B	Patient C	Patient D
**Penicillin allergy description**	Amoxicillin Reaction: hives	“Penicillins” Reaction: unknown	Penicillin Reaction: unknown	Penicillin Reaction: rash
	Date: unknown	Date: unknown	Date: unknown	Date: unknown
**Time to allergic reaction (h)**	78 h	32 h	<6 h	5 h
**Number of doses**	15	4	1	1
**Description of piperacillin-tazobactam reaction**	Rash to face, neck, and chest	Angioedema; right-sided face swelling and redness to the face	Not documented (Pt reported they experienced full body rash resembling “chemical burn” appearance)	Itchiness
**Confounding factors**	Tolerated first course piperacillin-tazobactam 2 wks prior on the same admission	Admitted with concurrent facial trauma (eye bruising)	Allergic reaction description is from the patient on subsequent admission	Patient reported itchiness (nonspecific)
**Immediate treatment?**	Yes × 1 dose of epinephrine	No	No	Yes × 1 dose of diphenhydramine
**Outcome of allergic reaction**	Immediate recovery/full resolution	Immediate recovery/full resolution	Immediate recovery/full resolution	Immediate recovery/full resolution
**High allergy alert medications w/in 48 h before/after**	Cefazolin and metronidazole <24 h from facial rash onset	Meropenem	Metronidazole and cephalexin (both within 6 h)	Cefazolin (within 24 h)
**Critical care ward Admission**	No	No	No	No
**Allergic reaction documented in EMR?**	Allergy documented in allergy profile	Allergic reaction not documented in allergy profile	Allergy documented in allergy profile	Allergy documented in allergy profile
**Naranjo score**	+3	+3	+5	+4
**Interpretation**	Possible adverse drug reaction	Possible adverse drug reaction	Probable adverse drug reaction	Possible adverse drug reaction

## Discussion

This study found that 98% of the 191 patients with a previously documented penicillin allergy tolerated one or more doses of piperacillin–tazobactam, demonstrating a low risk of cross-reactivity. Furthermore, majority of patients tolerated one or more doses of piperacillin–tazobactam across all risk stratification groups, including those in the “high risk anaphylactic group.”

The current literature regarding piperacillin–tazobactam use in patients with penicillin allergies has been centered around penicillin skin-testing or penicillin oral challenges in patients with previous allergies to piperacillin–tazobactam.^1–3^ These studies described low incidences of observed cross-reactivity, however these studies had small population sizes or consisted of case reports only. Comparably, this study assessed piperacillin–tazobactam at therapeutic doses in patients with previous penicillin allergies who had no prior penicillin skin-tests or penicillin oral challenges. Therefore, this study adds to the limited literature regarding the safety of using piperacillin–tazobactam in patients with labeled penicillin allergies, and the potential rates of cross-reactivity.

The risk stratification framework for this study was based on the Fraser Health Authority’s Antimicrobial Stewardship Program’s Risk Stratification for Penicillin Allergies guide which aligned with published guidelines at the time. Clinicians could risk stratify patients based on historical features, with lower risk patients considered for direct oral amoxicillin challenge as seen in the Drug Allergy: A 2022 Practice Parameter Update article.^12^ This practice has since been validated as safe and effective by the PALACE trial by Copaescu et al. (2023), where higher risk patients would undergo penicillin skin testing first, or alternative agents would be used based on risk of cross-reactivity.^13^ This study retrospectively assessed the use of piperacillin–tazobactam in patients with penicillin allergies which mimics the real-life scenarios where penicillin skin-testing cannot always be performed due to time constraints or resources.

Based on the results of this study, it is reasonable to consider using piperacillin–tazobactam in patients with penicillin allergies to limit the use of other broader-spectrum antibiotics. This includes patients with low-risk delayed reactions or poorly described symptoms, patients with previously documented anaphylactic allergic reactions, as well as patients with current unstable hemodynamics (Table 1). 90 of 91 patients in the “high risk” group tolerated piperacillin–tazobactam which accounts for approximately half of the study population, this included patients with previous anaphylactoid reactions and patients with unstable hemodynamics.

All 29 patients with a previously reported anaphylactic allergy or an anaphylactic-like reaction (shortness of breath, angioedema, swelling of unknown area) to penicillins tolerated one or more doses of piperacillin–tazobactam in this study. Although this is a small proportion of the larger study group (15%), it does suggest that piperacillin–tazobactam can be considered in patients with a history of a Type 1 hypersensitivity to penicillins. Additionally, just over a quarter of patients in this study were admitted to an intensive care or HAU during the time in which they received piperacillin–tazobactam. All patients in this group tolerated one or more doses of piperacillin–tazobactam, and no documentation of intolerance was noted. This highlights that it is reasonable to consider using piperacillin–tazobactam in patients with labeled penicillin allergies despite unstable hemodynamics. While the results of this study showed tolerability for patients with previous anaphylactic penicillin allergies and previous IgE-mediated allergies, a 1 or 2 step drug challenge can also be considered to provide reassurance that a full course of piperacillin–tazobactam would be tolerated by the patient.^12^

Although tolerance to piperacillin–tazobactam was observed for 100% of the patients with “well-documented delayed allergic reactions,” to penicillins, this category had only two patients. Therefore, an accurate assessment of piperacillin–tazobactam tolerability in patients with “well-documented delayed allergic reactions” to penicillins (Type II–IV hypersensitivity reactions) cannot be concluded from this study. Similar to previous studies investigating cross-reactivity between cephalosporins and carbapenems to penicillins, this study also found low-rates of cross-reactivity between penicillins and piperacillin–tazobactam, which supports the difference in side chains hypothesis.^17^ Of note, this study specifically examined the tolerance of piperacillin–tazobactam in patients with a history of penicillin allergies, excluding those with cephalosporin or carbapenem allergies, due to cephalosporins having different R1 side chains compared to aminopenicillins.^12^

Four of 191 patients (2%) were reported to not tolerate piperacillin–tazobactam (Table 3), however in each of those intolerance events (Patients A, B, C, and D) it is difficult to distinguish whether these were true allergic reactions to piperacillin–tazobactam, an allergic reaction to another antibiotic, or if their symptoms were related to their presenting disease states.

All four patients who experienced an intolerance to piperacillin–tazobactam had some diagnostic uncertainty as to whether they were true allergic reactions. Patient A previously tolerated piperacillin–tazobactam, which suggests that cefazolin or another confounding factor could have caused the rash. Patient B’s facial swelling and redness was confounded by concurrent facial trauma. Patient C had no documentation regarding the intolerance event in hospital and self-reported the allergy. Lastly, Patient D reported nonspecific itchiness without an observable rash or other symptoms. Therefore, it is difficult to distinguish if these intolerance events were a true allergic reaction to piperacillin–tazobactam or to another factor with overlapping characteristics or timing to the intolerance event. It is important to note that all four patients who reported intolerance event to piperacillin–tazobactam, did not experience a confirmed IgE-mediated allergic reaction after receiving piperacillin–tazobactam despite a previous labeled penicillin allergy. Per the Naranjo scoring tool, three were classified as possible ADR and one was classified as a probable ADR, which supports the notion that these were not definitive allergic reactions. Similar results were seen in the studies by Casimir-brown et al. (2021), Wong et al. (2021), and Gallardo et al. (2020) which also showed no pattern in cross-reactivity and low-rates of cross-reactivity during penicillin skin tests in patients with piperacillin allergies.

Overall, further research regarding the use of piperacillin–tazobactam in patients with previous penicillin allergies is required, specifically in patients with skin-test confirmed penicillin allergies which would represent patients with true penicillin allergy. In addition, further research is needed to assess piperacillin–tazobactam tolerability in patients with type II-IV hypersensitivities reactions to penicillins as this study was only able to enroll two patients in this category.

### Limitations

It is important to note that this study does have some limitations. Firstly, it is difficult to determine if patients had a true penicillin allergy given the poor documentation of prior allergic symptoms and time frame. Majority of the study population had an unknown amount of time since their initial penicillin allergic reaction (73%), or the reaction had occurred greater than 10 years ago (20%). However, this study is pragmatic in design and aimed to reflect real-world practices, as prescribers are often faced with the same uncertainty. Secondly, there is the risk of potentially under-recognizing delayed reactions beyond hospitalization as patients were not followed after discharge. Lastly, it is difficult to assess piperacillin–tazobactam intolerability based on the documentation available, given other confounding factors which may have overestimated the tolerability rate in our study. While the above limitations may affect some outcomes of this study, the data does support the use of piperacillin–tazobactam in patients with previous documented penicillin allergies.

### Conclusion

Overall, the results of this study showed a low risk of cross-reactivity between piperacillin–tazobactam and other penicillin antibiotics. Prior documented penicillin allergies should not be an absolute contraindication to using piperacillin–tazobactam in patients and piperacillin–tazobactam should be considered to minimize carbapenem and other broad-spectrum antibiotics. The results of this study can support antimicrobial stewardship interventions, such as helping to guide empiric decision-making protocols, or by further supporting guidelines in highlighting the low risk of cross-reactivity between piperacillin–tazobactam and penicillins. Further studies are needed to assess the incidence of cross-reactivity in patients with skin-test confirmed penicillin allergies on a larger scale and in a prospective setting, to minimize confounding factors.
